# Advanced Machine Learning Models for High-Temperature Magnetoresistivity Predictions of Ni_81_Fe_19_ Monolayers

**DOI:** 10.3390/nano16010051

**Published:** 2025-12-30

**Authors:** Tarik Akan, Perihan Aksu, Recep Sahingoz, Feliks S. Zaseev, Vladislav B. Zaalishvili, Tamerlan T. Magkoev

**Affiliations:** 1 Physics Department, Yozgat Bozok University, 66100 Yozgat, Türkiye; tarik.akan@bozok.edu.tr (T.A.); recep.sahingoz@bozok.edu.tr (R.S.); 2 Institute of Nanotechnology, Gebze Technical University, 41400 Kocaeli, Türkiye; paksu@gtu.edu.tr; 3 Nanomagnetism Research and Application Center, Gebze Technical University, 41400 Kocaeli, Türkiye; 4 Institute of Electronics and Telecommunications, Peter the Great Saint Petersburg Polytechnic University, Polytechnicheskaya 29, 195251 Saint-Petersburg, Russia; zaseev.feliks@mail.ru; 5 Geophysical Institute—The Affiliate of Vladikavkaz Scientific Centre of the Russian Academy of Sciences, Markova 93a, 362002 Vladikavkaz, Russia; vzaal@mail.ru; 6 Laboratory of Physics of Adsorption Phenomena, Department of Condensed Matter Physics, North Ossetian State University, Vatutina 44-46, 362025 Vladikavkaz, Russia

**Keywords:** magnetism, nanomaterials, machine learning

## Abstract

A 5 nm thick polycrystalline $Ni_{81}Fe_{19}$ film was sputter-deposited onto a circular 3-inch diameter, 390 μm thick single-crystal wafer with $SiO_{2}$ surface layers. The magnetoresistance (MR) of the sample was analyzed as a function of applied DC magnetic field and temperature using the Van der Pauw technique. Magnetic measurements were carried out over a temperature range of 25 °C to 350 °C using a Lake Shore Hall Effect Measurement System (HEMS). An external magnetic field ranging from +14 kG to −14 kG was applied at each temperature value to observe changes in resistance. Hall coefficients and resistance were obtained by applying current in both directions with different contact configurations. Machine learning techniques, including Random Forest Regression, were employed to predict magnetoresistivity beyond 350 °C; the best-performing model achieved $R^{2}$ values up to 0.9449 with MSE as low as 0.0071, and enabled Curie temperature estimation with $T_{C}\approx 590.97\;{}^{\circ}C\;$. This study highlights the potential of machine learning in accurately forecasting material properties beyond experimental limits, providing enhanced predictive models for the magnetoresistive behavior and critical temperature transitions of $Ni_{81}Fe_{19}$ .

## 1. Introduction

Magnetoresistive sensors are essential components in high-density information storage and position/speed monitoring technologies [1]. Their performance is influenced not only by the field dependence of the material parameters but also by the geometric configuration of the device [2]. Narrow band-gap semiconductors such as InSb have attracted considerable interest due to their high carrier mobilities, which provide superior sensitivity and extended frequency response beyond the 10–20 kHz range typical of Si Hall sensors [3]. $Ni_{81}Fe_{19}$ (Permalloy) is widely employed in spin-valve read heads because of its negligible magnetostriction, which effectively minimizes stress-induced anisotropy [4,5]. In ferromagnetic metals like $Ni_{81}Fe_{19}$, electrical resistivity is strongly influenced by spin-dependent scattering processes. The likelihood of such scattering depends on spin orientation and magnetic ordering [6], giving rise to the anisotropic magnetoresistance (AMR) effect. When magnetization is aligned by an external field, spin disorder is reduced, resulting in lower electron scattering and decreased resistivity. In contrast, at zero field, a demagnetized multi-domain state enhances spin-disorder scattering, leading to higher resistivity. Temperature introduces additional effects: as it rises, thermal spin disorder increases resistivity, and near the Curie temperature ($T_{C}$), critical spin fluctuations produce a pronounced peak [7]. This phenomenon—often described as enhanced spin-disorder scattering in the critical region—is analogous to the Curie–Weiss behavior of magnetic susceptibility. Above $T_{C}$, susceptibility follows the Curie–Weiss law, reflecting the loss of long-range ferromagnetic order. Below $T_{C}$, spontaneous magnetization decreases progressively with temperature and vanishes at $T_{C}$ through a second-order phase transition. This temperature dependence, sometimes approximated by mean-field models such as $M(T)=1-{\left(T/T_{C}\right)}^{\alpha }$ provides the theoretical framework for interpreting our high-temperature magnetotransport data.

In practice, extending magnetoresistivity characterization to elevated temperatures is challenging: measurements may be limited by instrument constraints and affected by thermal drift, contact-resistance changes, or irreversible modifications in ultrathin films (e.g., oxidation or interdiffusion). Moreover, the high-temperature response reflects multiple competing scattering mechanisms, including spin-disorder contributions that grow with temperature and can become strongly non-linear near the Curie region. These factors make straightforward extrapolation beyond the experimentally accessible range unreliable and motivate data-driven surrogate models for robust high-temperature prediction.

Despite significant advances in magnetoresistive technology, accurately predicting material properties, such as magnetoresistivity at elevated temperatures, remains a challenge [8]. Traditional experimental methods often face limitations when extending observations beyond experimentally feasible conditions [9]. In recent years, machine learning (ML) has emerged as a powerful tool to address these challenges, enabling data-driven predictions that extend beyond the limits of experimental data [10,11,12,13].

The novelty of the present work is to treat high-temperature magnetoresistivity prediction as a supervised learning problem based on the full field-dependent curves, rather than predicting only a few scalar summaries. We benchmark multiple regression models under a unified evaluation protocol, then use the best-performing model to extrapolate magnetoresistivity to temperatures above the measurement limit. Importantly, the extrapolated temperature dependence is also used to estimate the Curie temperature, and the predicted behavior is checked for physical consistency using normalized representations (e.g., trends versus $T/T_{C}$). This combined curve-level prediction, extrapolation, and $T_{C}$ estimation provides a device-relevant surrogate modeling workflow that reduces the need for technically challenging high-temperature experiments.

This study leverages advanced machine learning models, including Support Vector Regression (SVR), Random Forest Regression (RFR), and Neural Networks (NN), to analyze and predict the magnetoresistive behavior of $Ni_{81}Fe_{19}$ (Permalloy) monolayers. By employing these sophisticated models, we aim to generate accurate predictions of magnetoresistivity at temperatures exceeding 350 °C, a range that is typically inaccessible through conventional experimental methods [14,15]. The integration of ML models in our approach not only enhances predictive accuracy but also enables more precise estimation of critical material properties, such as the $T_{C}$ [16,17]. These predictive insights are expected to play a crucial role in advancing the application of Permalloy in next-generation technologies, including high-density information storage and spintronic devices.

## 2. Materials and Methods

### 2.1. Experimental Techniques

The 5 nm-thick $Ni_{81}Fe_{19}$ (Permalloy) films were deposited onto a 3-inch single-crystal Si wafer with its native $SiO_{2}$ oxide layer preserved (prepared in-house by Recep Sahingoz, Yozgat Bozok University, Yozgat, Türkiye.). Films with an individual thickness of 5 nm are deposited under high-vacuum conditions using magnetron sputtering. The as-deposit films were 50–60 mm long and 10 mm wide. The samples were thoroughly cleaned with acetone and alcohol prior to further processing to remove surface impurities.

The films were then cut into $5\times 5\;mm^{2}$ pieces for measurement. Electrical contacts were established by coating the sample corners with silver paint and attaching copper wires [18]. Resistance was measured using the Van der Pauw technique, where a constant current (10–100 mA) was applied in-plane via a constant current source, and the corresponding voltage was measured using a digital voltmeter. The Helmholtz coils were driven by a bipolar power supply to generate the magnetic field.

**Figure 1:**
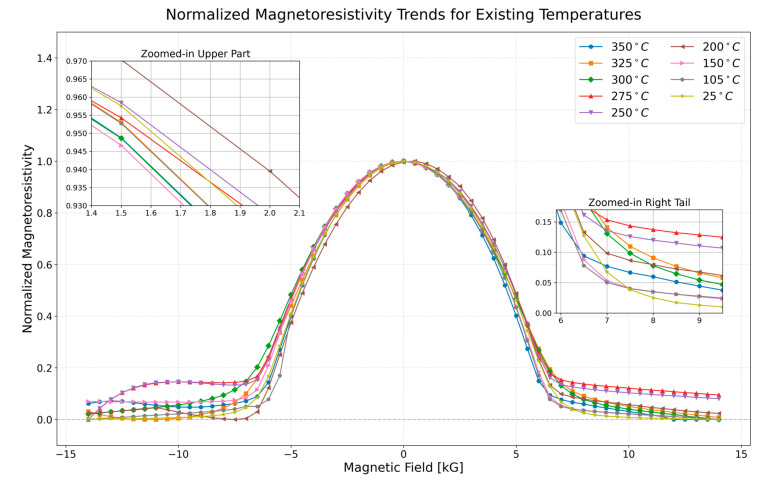
This plot illustrates the normalized magnetoresistivity trends of $Ni_{81}Fe_{19}$ at various existing temperatures ranging from 25 °C to 350 °C. The data shows the dependence of magnetoresistivity on the applied magnetic field across these temperatures. The zoomed-in sections highlight specific regions where differences in magnetoresistivity behavior.

Magnetic measurements were performed using the Lake Shore Hall Effect Measurement System (HEMS) in 30 steps over the temperature range of 25 °C to 350 °C [19]. Hall coefficients and resistance at each temperature were obtained by reversing the current direction and using all possible contact configurations [20,21]. Temperature-dependent magnetic measurements were carried out on 5 nm-thick $Ni_{81}Fe_{19}$ (Permalloy) films deposited under vacuum on $SiO_{2}$ [22,23]. An external magnetic field of was applied to the sample at each temperature. Resistance changes with applied magnetic field and temperature were measured at each temperature, as shown in Figure 1.

In Figure 1, temperatures were kept constant during the experiment. For example, magnetoresistance measurements were made by changing the magnetic field, while the temperature was fixed at 250 °C [24]. It can be seen in the normalized graphs that the magnetoresistivity values show considerable differences [25]. It is well known that changes in resistivity arise from the material’s composition, geometry, and external factors such as temperature, stress, and pressure, as discussed in similar studies [26]. The temperature was kept constant during the application of the magnetic field. Separate magnetic measurements were taken for each temperature value and presented in the same graph [27].

The magnetization of the material is inversely proportional to the temperature. Magnetization decreases with temperature and disappears at the $T_{C}$, where the material becomes paramagnetic. This phenomenon is known as the Curie–Weiss law in ferromagnetic materials [9]. Even though the sample temperature is constant under the applied magnetic field, the observed resistance change is due to changes in Hall mobility and carrier density [5].

In the absence of a magnetic field, the apparent carrier density decreases with increasing temperature, whereas the Hall mobility increases at higher temperatures. At 25 °C,  carrier density is approximately $3\times 10^{21}\;cm^{-3}$; this falls to $\sim 1.6\times 10^{21}\;cm^{-3}$ by 350 °C. Meanwhile, the Hall mobility rises from $75\;cm^{2}/V\cdot s$ at 250 °C to $96\;cm^{2}/V\cdot s$ at 350 °C [28]. These characteristics differ from those typical in ordinary metals (where carrier density is approximately stable and the Hall mobility typically decreases with higher temperature owing to phonon scattering). Instead, these behaviors arise from the interplay of the ordinary and anomalous Hall effects (AHEs) in ferromagnetic $Ni_{81}Fe_{19}$. When the AHE contribution (scaling with magnetization) dominates, it counteracts the ordinary Hall voltage, resulting in an apparent overestimation of carrier density.

When the temperature rises while the magnetization also falls, the effect of the AHE is minimized, exposing the intrinsic carrier density which seems to decline. The increase in μ is mainly attributed to the remaining carriers experiencing reduced scattering in the near-paramagnetic regime, even though phonon scattering increases.

In summary, the observed decrease in carrier density and increase in the Hall mobility at elevated temperatures are explained by the weakening anomalous Hall contribution and prevailing scattering mechanisms, rather than actual variations in carrier density or reduced phonon scattering.

### 2.2. Machine Learning Methodology

This study applies several machine learning (ML) models, including Support Vector Regression (SVR), Random Forest Regression (RFR), Decision Tree Regression, Neural Networks (NN), Gaussian Process Regression (GPR), and Linear Regression, to predict magnetoresistivity and estimate critical temperatures such as the $T_{C}$. These models are selected for their ability to model non-linear relationships and to extend predictions beyond the experimental temperature range. This section details the architectures, key parameters, configurations for each model, and the crucial role of synthetic data generation.

SVR captures non-linear dependencies in the data using a radial basis function (RBF) kernel. The model is configured with three key hyperparameters: the regularization parameter (C), the kernel coefficient (γ), and the ϵ-insensitive margin. Here, based on systematic tuning, the hyperparameters C=10, kernel width γ=0.1, and ϵ=0.01 were selected. Specifically, grid searches and cross-validation were performed: a lower C (e.g., 1) was observed to underfit (high bias, producing smoother predictions that missed curvature), whereas a very high C (e.g., 100) overfit the noise (high variance, capturing small fluctuations that hurt generalization). C=10 gave the best balance between bias and variance on validation sets. For the kernel parameter, we found γ=0.1 optimal; a larger γ (such as 1.0) made the SVR local, fitting individual points tightly but failing to generalize (similar to overfitting), while a much smaller γ (e.g., 0.01) made it global (almost linear). The chosen γ=0.1 allowed the SVR to capture the broad non-linear trend without chasing high-frequency noise. The parameter ϵ, controlling the tolerance for the fit error, was set to ϵ=0.01 (for normalized resistivity values ~0–1), allowing minor deviations to be ignored and improving robustness. Using a significantly larger ϵ (e.g., 0.1) slightly degraded accuracy (as the model would ignore small but systematic variations), while a smaller ϵ (e.g., 0.001) did not noticeably improve accuracy but made training slower. With these optimized hyperparameters, the SVR achieved prediction accuracy almost on par with RFR.

RFR operates as an ensemble learning technique, constructing 400 decision trees trained on bootstrapped subsets of data and features. A distinct prediction is made for each tree.

Inference is determined by the ensemble output, which is defined as the average prediction from all the trees. This process is able to effectively preserve complex non-linear interactions between magnetoresistivity and magnetic field while reducing model variability and preventing overfitting. With the noise-augmented training scheme as seen above, the relatively high number of estimators (n=400) ensures the model stability against noisy inputs.

In contrast, in decision tree regression, the input data is compartmentalized using a single hierarchical structure through the iterative selection of optimal feature thresholds. This methodology is characterized by high interpretability and clearly defined decision logic, making it suitable for exploratory analysis. However, when applied to small or moderately sized datasets, it is particularly prone to overfitting, resulting in the capture of noise rather than the true signal. Consequently, while decision trees may demonstrate efficacy in training data, their capacity for generalization to unseen data is frequently suboptimal. Notwithstanding this limitation, decision tree regression remains a valuable baseline for comparing the performance of more robust ensemble methods, such as random forests.

In order to learn non-linear mappings from magnetic field to magnetoresistivity, NN were implemented as deep feedforward architectures. Four dense layers make up the enhanced NN architecture: 512 neurons for the input layer, 256 and 128 neurons for the hidden layers, and an output layer at the end. Dropout layers with rates of 0.4 and 0.3 are used to avoid overfitting, and all hidden layers introduce non-linearity using the ReLU activation function. To stabilize training, batch normalization is added after the initial dense layer. In order to minimize the mean squared error loss, the model is trained using the Adam optimizer at a learning rate of 0.0001. To avoid overfitting and enhance generalization, adaptive learning rate reduction (factor = 0.6, patience = 150) and early stopping (patience = 200) are used.

GPR offers a probabilistic modeling approach that can not only provide point predictions but also measures of uncertainty. Hyperparameters were optimized with up to 30 restarts to overcome local minima in the likelihood landscape. Normalized targets were utilized to learn the model that was regularized with an α=0.001 noise term. GPR demonstrated the ability to interpolate within the training range and supplied predictive uncertainty where it is extrapolated that rendered the method highly useful for the calculation of confidence intervals in sparse or noisy spaces.

Linear Regression serves as a baseline model for evaluating the linearity of the relationship between temperature, magnetic field, and magnetoresistivity. While computationally efficient, it is insufficient to capture the complex, non-linear interactions in the dataset, highlighting the need for more advanced techniques.

Synthetic data generation plays a critical role in enhancing the robustness and generalizability of the machine learning models applied in this study. Gaussian noise is introduced to the experimental data to simulate the variability often encountered in real-world measurements. Additionally, synthetic magnetoresistivity values are computed for temperatures beyond the experimental range using the magnetization model $M(T)\;=\;1\;-\;{\left(T/T_{C}\right)}^{\alpha }$, where $T_{C}$ and α are fitted parameters derived from the experimental data. These model predictions are further smoothed using Gaussian filtering to minimize abrupt variations while retaining key trends. This approach ensures that the ML models train on a broader spectrum of conditions, facilitating improved extrapolation and predictive accuracy for unseen temperature and magnetic field values. After augmentation, the training pool contained 50% synthetic data.

The dataset consists of 300 measurements collected in a temperature range of 25 °C to 350 °C and a magnetic field of ±14 kG. These data points are divided into 70% for training, 15% for validation, and 15% for testing. Cross-validation is performed to ensure model generalizability, and hyperparameter optimization is performed for SVR, RFR, NN, and GPR to fine-tune their performance. Although the dataset is modest, the magnetoresistivity exhibits a structured response for each temperature it is approximately Gaussian like in the intermediate field range, while showing clear and systematic deviations in the high field tail. Moreover, the temperature evolution follows a physically constrained trend that can be approximated by $M(T)=\;1-{\left(T/T_{C}\right)}^{\alpha }$, which supports stable learning and enables estimation of $T_{C}$. This structure reduces the amount of data required to learn robust patterns, but the tail discrepancies indicate that a single simple parametric form is insufficient across the full field range. Therefore, ML is employed to learn a global surrogate model that captures both Gaussian like core and the non-Gaussian tail behavior, and to support extrapolation beyond the experimental temperature limit.

Although many regression algorithms exit, we intentionally focus on a compact set of widely used approaches that cover complementary modeling strategies. This supports the main aim of the paper, which is to perform a controlled comparison under identical preprocessing, data splitting, and evaluation metrics. For reproducibility, all hyperparameters and training settings are reported so that readers can regenerate the results. The next section presents the comparative performance and discusses the predictive behavior of each method.

## 3. Results and Discussion

**Figure 2:**
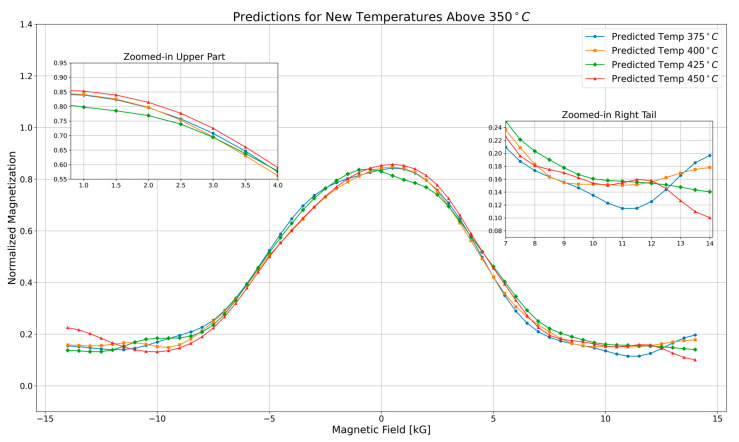
Predictions for new temperatures above 350 °C. This plot shows the predicted magnetoresistivity for temperatures above 350 °C The zoomed-in sections highlight the upper part and right tail of the curve.

The behavior of the MR data, as shown in Figure 2, demonstrates an almost perfect Gaussian pattern in the central and intermediate regions of the magnetic field range. This consistency underscores the quality of the dataset and the robustness of the machine learning models. However, in the tail regions, discrepancies between the magnetoresistivity curves become more pronounced for different temperature values, which is a common feature in magnetic systems as they approach magnetic saturation.

As magnetic saturation effects dominate in the tail regions, the magnetoresistive sensitivity is reduced, leading to the observed deviations. These deviations do not undermine the overall fit or predictive accuracy of the models in the primary and intermediate regions but rather highlight the physical limitations and complexities of magnetoresistive behavior at extreme magnetic field values. This transition from regular Gaussian behavior to saturation effects provides valuable insights into the material’s magnetoresistive properties, which are critical for understanding its performance across different regimes.

To extend the understanding of the material’s properties beyond the experimentally measured range, ML techniques were employed to analyze the dataset of magnetoresistivity as a function of temperature and magnetic field. The dataset, comprising measurements across a temperature range of 25–350 °C and magnetic fields up to 14 kG, was used to train several ML models [10]. These models included regression techniques, which were trained to predict magnetoresistivity beyond the experimental limits [8].

The ML models were evaluated using metrics such as Mean Absolute Error (MAE) and the $R^{2}$ score to assess predictive accuracy [29]. To summarize the temperature-wise robustness of the two best-performing models, we report the average and standard deviation of their scores across the temperature steps listed in Table 1 and Table 2. Over this range, Random Forest Regressor achieves an average $R^{2}$ score of 0.885±0.036, while Support Vector Regressor yields 0.885±0.023; consistently, the average MSE values in Table 1 are 0.0164±0.0062 for Random Forest and 0.0169±0.0041 for SVR. Since the magnetoresistivity curves are dominated by a Gaussian-like profile, even small gains in overall agreement are valuable for preserving the full curve shape; therefore, Random Forest is adopted as the preferred model for high-temperature extrapolative predictions beyond the experimental window [30,31].

The predictions plot in Figure 2 illustrates the variation in normalized magnetization as a function of the magnetic field at temperatures exceeding 350 °C (375 °C, 400 °C, 425 °C, and 450 °C). Each curve represents a specific temperature and follows a Gaussian-like profile, peaking at zero magnetic field and symmetrically decreasing as the field strength increases. As temperature increases, magnetization decreases, which is expected due to thermal agitation reducing magnetic alignment. The 450 °C curve (red) consistently exhibits the lowest magnetization values, while the 375°C curve (blue) maintains the highest values across the field range.

The zoomed-in upper region, covering the 1.7 kG to 4.5 kG range, provides a closer look at how magnetization decreases with increasing magnetic field. The curves follow a smooth downward trend, with higher-temperature curves lying below lower-temperature ones. This gradual separation of the curves confirms the expected temperature-dependent decline in magnetization, with no abrupt changes in behavior.

The zoomed-in right tail, spanning 10 kG to 14 kG, highlights magnetization behavior at high magnetic fields. In this region, magnetization values are generally low, but temperature-driven variations cause slight deviations from the Gaussian shape. The 450 °C curve (red) reaches the lowest magnetization values, reflecting stronger thermal effects at higher temperatures. Despite these differences, all curves stabilize at low magnetization values, suggesting a reduced sensitivity to the magnetic field at elevated temperatures, where thermal fluctuations dominate.

Using the refined models, predictions were made for magnetoresistivity at temperatures significantly higher than 350 °C, specifically at 375 °C, 400 °C, 425 °C, and 450 °C. These predictions provide insights into the behavior of the material at higher temperatures, which are challenging to achieve in standard laboratory conditions [32]. The plots reveal that as the temperature increases beyond 350 °C, the predicted magnetoresistivity values continue to follow the expected trend, demonstrating the models’ capability to extrapolate beyond the experimentally available data [33].

**Figure 3:**
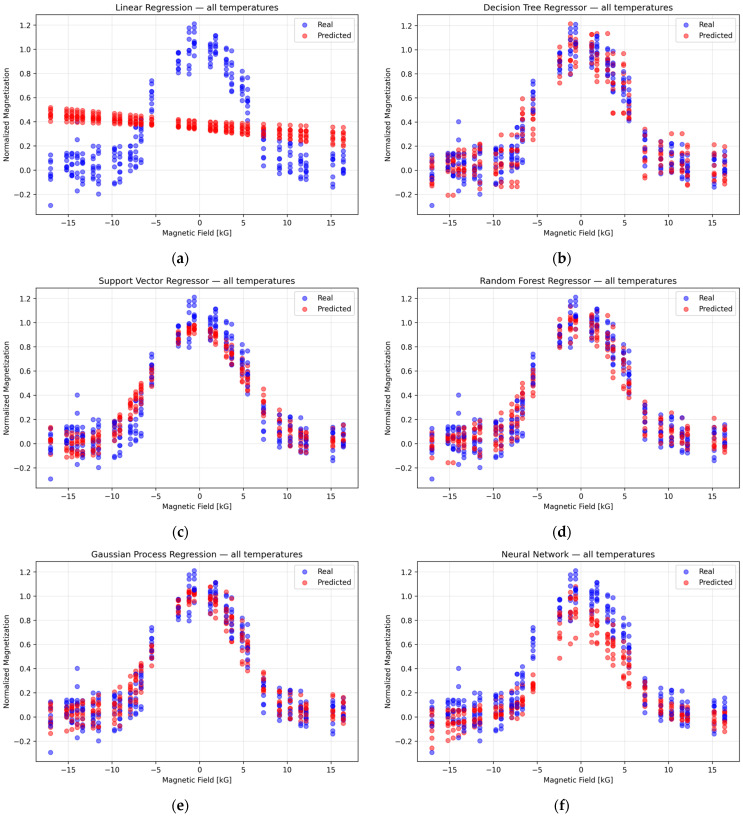
Real (blue) vs. Predicted (red) values at 25 °C shows the different ML models. Subplots correspond to: (**a**) Linear Regression, (**b**) Decision Tree Regressor, (**c**) Support Vector Regressor, (**d**) Random Forest Regressor, (**e**) Gaussian Process Regression, and (**f**) Neural Network.

The real vs. predicted values for different ML models at 25 °C are shown in Figure 3. This comparison highlights the performance of various models, with the RFR model providing the most accurate predictions. The scatter plot demonstrates a strong alignment between the predicted and actual values for the RFR, while other models show larger deviations, indicating the superior predictive power of the Random Forest model in this scenario [34,35,36].

Importantly, the Random Forest Regression (RFR) model did not merely achieve high predictive accuracy—it also captured meaningful physical trends inherent in the data. Specifically, the model recognized that increasing the temperature generally raises the material’s baseline resistivity, a consequence of enhanced scattering at higher T, while applying a higher magnetic field lowers the resistivity by suppressing magnetic scattering. In other words, it successfully reflected the well-known magnetoresistive behavior where an external field reduces resistivity (negative magnetoresistance) by aligning magnetic moments and thereby reducing spin-disorder scattering [37].

These trends are consistent with established physics: near the $T_{C}$ (where spins become disordered), one typically observes a strong negative magnetoresistance (i.e., a significant drop in resistivity under applied field), whereas at low temperatures (deep in the ferromagnetic phase with ordered spins), the magnetoresistive effect is much weaker [37].

The key point is that the RFR’s predictions respect known physical behavior (e.g., no unphysical spikes or trends), indicating that the model learned underlying dependencies rather than spurious correlations. In effect, the RFR operates as an implicit physics-based model for resistivity as a function of temperature and field. Its learned mapping can be interpreted as effectively capturing separate contributions of temperature and magnetic field, for instance:

(1) $$R\left(T,H\right)\approx R_{base}\left(T\right)+\Delta R_{mag}(T,H),$$

where $R_{base}(T)$ is a baseline resistivity increasing with temperature (reflecting temperature-dependent scattering processes), and $\Delta R_{mag}(T,\;H)$ is a field-dependent component associated with spin-disorder scattering, which decreases as the field H increases.

Physically, $R_{base}(T)$ represents the resistivity in the absence of magnetic scattering (e.g., due to lattice or impurity scattering), while $\Delta R_{mag}(T,\;H)$ represents the additional resistivity from spin disorder, which is suppressed by an external magnetic field [38]. This form is analogous to Matthiessen’s rule—where total resistivity is approximately the sum of independent contributions—and mirrors what is observed in magnetoresistive materials.

Near the $T_{C}$, the spin-disorder contribution $\Delta R_{mag}(T,\;H)$ is large (random spins increase resistivity), but a strong enough magnetic field can align the spins and drastically reduce this part of the resistivity [37]. Far below $T_{C}$, the spins are mostly aligned even at zero field, so $\Delta R_{mag}(T,\;H)$ is inherently small, and the application of a field yields only a minor resistivity change, consistent with the weaker magnetoresistance observed at low T.

Although this two-component form was not explicitly imposed in the modeling, it was effectively discovered from the training data by the RFR (and, to a lesser extent, the SVR), reflecting the underlying physical relationship. This accounts for the model’s high predictive accuracy and the fact that its predictions conform to established physical laws of magnetoresistivity, thereby lending credibility to its use in analyzing and predicting magnetoresistive behavior.

**Figure 4:**
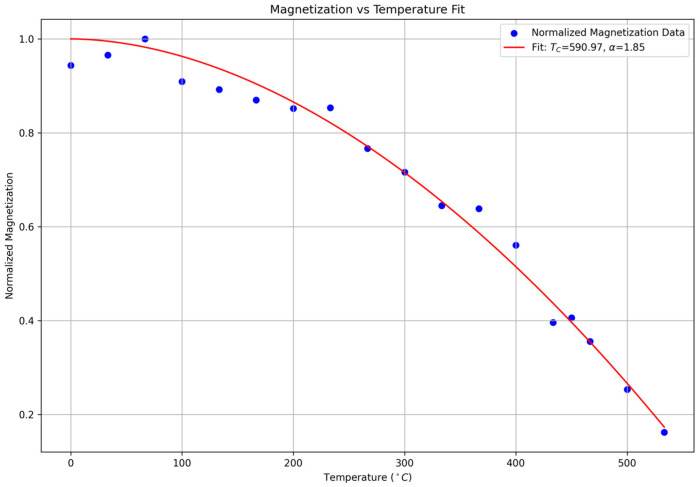
Magnetoresistivity vs. Temperature Fit. This plot shows the magnetoresistivity data as a function of temperature with a fitted curve, indicating the trend and behavior of magnetoresistivity.

After identifying RFR as the best-performing model, predictions were extended to temperatures above 350 °C, beyond the experimental range. Using the predicted magnetoresistivity data, $T_{C}$ was determined by fitting the data to the equation $M(T)\;=\;1\;-\;{\left(T/T_{C}\right)}^{\alpha }$, where α is a fitting parameter. Through this fitting procedure (see Figure 4), the $T_{C}$ was estimated to be approximately 590.97°C demonstrating the success of our methodology and the quality of both the data and model predictions. This result aligns closely with recent studies that report a $T_{C}$ of 581°C for $Ni_{81}Fe_{19}$ [17], underscoring the precision of the RFR model in predicting critical material properties.

**Figure 5:**
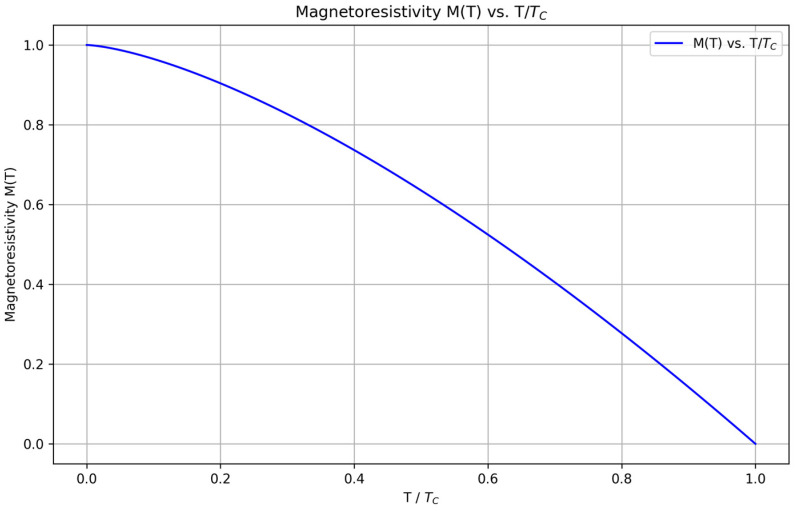
Magnetoresistivity M(T) vs. $T/T_{C}$. The plot shows the normalized magnetoresistivity as a function of the normalized temperature, comparing well with theoretical models and existing literature [40]. This validation against known behaviors underscores the accuracy of the machine learning predictions.

The normalized magnetoresistivity M(T) as a function of the normalized temperature $T/T_{C}$ is shown in Figure 5 [39,40]. This plot is particularly significant as it compares well with the theoretical models and existing literature, indicating that the ML predictions align closely with known physical behaviors [40,41]. The curve demonstrates a smooth decline in magnetoresistivity with increasing normalized temperature, consistent with theoretical expectations, and further validates the model’s accuracy in capturing the material’s intrinsic properties [36].

Overall, this advanced analysis provided enhanced predictive models for the material’s magnetoresistive behavior and critical temperature transitions [24]. The Random Forest Regression model, in particular, showcased the potential of machine learning in accurately forecasting material properties beyond experimental temperature limits [11]. The extension of predictions to elevated temperatures underscores the effectiveness of these ML models in offering insights into material behavior under conditions that are challenging to replicate experimentally. This study underscores the significant role that machine learning can play in advancing our understanding of complex material properties and aiding in the development of new technologies [42].

## 4. Conclusions

This study has demonstrated the significant potential of advanced machine learning techniques in predicting magnetoresistive behavior in $Ni_{81}Fe_{19}$ thin films of 5 nm at elevated temperatures. By successfully applying models such as Support Vector Regression, Random Forest Regression, and Neural Networks, a robust framework for understanding and predicting material properties beyond conventional experimental limits has been established [43]. The Random Forest Regression model emerged as the most effective, delivering the lowest Mean Absolute Error (MAE) and highest $R^{2}$ scores, which indicates high predictive accuracy for magnetoresistivity across a range of temperatures [44]. This suggests that Random Forest Regression is particularly well-suited for handling complex datasets and extracting meaningful insights regarding the material’s behavior [44,45].

The ability of the machine learning models to estimate the $T_{C}$ with precision further highlights their potential for identifying critical transitions in magnetic properties [30]. These findings provide a foundation for future research and development in materials science, particularly in designing high-performance materials for applications in spintronic devices and high-density information storage [46]. These predictions can support sensor and device design by estimating high-temperature response trends, helping define operating windows and expected degradation behavior without requiring additional high-temperature measurements. The study underscores the transformative role of machine learning in materials science, offering new avenues for predicting and understanding complex material behaviors. By extending predictions beyond experimentally accessible conditions, machine learning models can significantly accelerate the development of advanced materials and technologies [23].

Future work will focus on refining these models further and exploring their applicability to other magnetic materials, potentially broadening the scope and impact of machine learning in predicting and enhancing material properties [47] such as mobility and carrier density. The continued integration of machine learning techniques in material science research promises to unveil new insights and drive technological advancements in various fields [48].

## Figures and Tables

**Table 1:** MSE Scores of Different Models for Various Temperatures (°C).

ML Models	Temperature (°C)								
	25	105	150	200	250	275	300	325	350
Linear Regression	0.1589	0.1929	0.1642	0.1452	0.1328	0.1514	0.1435	0.1664	0.1747
Decision Tree Regressor	0.0283	0.0243	0.0144	0.0098	0.0261	0.0244	0.0199	0.0298	0.0258
Support Vector Regressor	0.0137	0.0235	0.0116	0.0126	0.0172	0.0200	0.0136	0.0172	0.0196
Random Forest Regressor	0.0160	0.0239	0.0071	0.0078	0.0187	0.0174	0.0149	0.0221	0.0192
Gaussian Process Regression	0.0288	0.0502	0.0243	0.0295	0.0595	0.0339	0.0327	0.0357	0.0312
Neural Network	0.0282	0.0558	0.0199	0.0274	0.0370	0.0545	0.0737	0.0405	0.0317

**Table 2:** $R^{2}$ Scores of Different Models for Various Temperatures (°C).

ML Models	Temperature (°C)								
	25	105	150	200	250	275	300	325	350
Linear Regression	−0.1529	−0.1001	−0.1256	−0.0423	−0.1022	−0.0697	−0.0833	−0.1148	−0.0986
Decision Tree Regressor	0.7941	0.8615	0.9013	0.9301	0.7835	0.8276	0.8501	0.7991	0.8379
Support Vector Regressor	0.9008	0.8657	0.9207	0.9098	0.8572	0.8651	0.8972	0.8842	0.8765
Random Forest Regressor	0.08578	0.8638	0.9334	0.9449	0.8452	0.8770	0.8874	0.8521	0.8789
Gaussian Process Regression	0.7914	0.7141	0.8335	0.7880	0.5066	0.7606	0.7535	0.7554	0.8037
Neural Network	0.7954	0.6819	0.8637	0.8031	0.6928	0.6149	0.4436	0.7287	0.8003

## Data Availability

The data supporting the findings of this study are available from the corresponding author upon reasonable request.

## References

[1] Solin S.A., Thio T., Hines D.R., Heremans J.J. (2000). Enhanced room-temperature magnetoresistance in inhomogeneous magnetic multilayers. Science.

[2] Wieder H.H. (1971). Hall Generators and Magnetoresistors.

[3] Chang Y., Wang Y., Zhang J., Xing Y., Li G., Deng D., Liu L. (2022). Overview on the design of magnetically assisted electrochemical biosensors. Biosensors.

[4] Gurney B.A., Carey M., Tsang C., Williams M., Parkin S.S.P., Fontana R.E., Grochowski E., Pinarbasi M., Lin T., Mauri D. (2005). Spin valve giant magnetoresistive sensor materials for hard disk drives. Ultrathin Magnetic Structures IV: Applications of Nanomagnetism.

[5] Biondo A., Nascimento V.P., Lassri H., Passamani E.C., Morales M.A., Mello A., De Biasi R.S., Baggio-Saitovitch E. (2004). Structural and magnetic properties of Ni81Fe19/Zr multilayers. J. Magn. Magn. Mater..

[6] Valenzuela S., Gambardella P., Garello K., Klein O., Sierra J., Sinova J. (2024). Spintronic materials. Encyclopedia of Condensed Matter Physics.

[7] Zhao Q., Sun K., Wu S., Li H.F. (2025). Crystal, ferromagnetism, and magnetoresistance with sign reversal in a EuAgP semiconductor. J. Mater..

[8] Pecharsky V.K., Gschneidner K.A. (1997). Giant magnetocaloric effect in Gd5(Si2Ge2). Phys. Rev. Lett..

[9] Brown G.V. (1976). Magnetic heat pumping near room temperature. J. Appl. Phys..

[10] Stanev V., Oses C., Kusne A.G., Rodriguez E., Paglione J., Curtarolo S., Takeuchi I. (2018). Machine learning modeling of superconducting critical temperature. npj Comput. Mater..

[11] Court C.J., Cole J.M. (2020). Magnetic and superconducting phase diagrams and transition temperatures predicted using text mining and machine learning. npj Comput. Mater..

[12] Möller J.J., Körner W., Krugel G., Urban D.F., Elsässer C. (2018). Compositional optimization of hard-magnetic phases with machine-learning models. Acta Mater..

[13] Li W., Lan X., Liu X., Zhang E., Deng Y., Wang K. (2022). Switching plasticity in compensated ferrimagnetic multilayers for neuromorphic computing. Chin. Phys. B.

[14] Dey C., Yari P., Wu K. (2023). Recent advances in magnetoresistance biosensors: A short review. Nano Futures.

[15] Yoon S.S., Kim D.Y. (2023). Permeability measurement performance of PHR sensor according to direction of driving current. J. Korean Magn. Soc..

[16] Liaw A., Wiener M. (2002). Classification and regression by randomForest. R News.

[17] Yamanoi K., Semizu H., Nozaki Y. (2022). Enhancement of room-temperature unidirectional spin Hall magnetoresistance by using a ferromagnetic metal with a low Curie temperature. Phys. Rev. B.

[18] James G., Witten D., Hastie T., Tibshirani R. (2017). An Introduction to Statistical Learning.

[19] Jain A., Ong S.P., Hautier G., Chen W., Richards W.D., Dacek S., Cholia S., Gunter D., Skinner D., Ceder G. (2013). The Materials Project: A materials genome approach to accelerating materials innovation. APL Mater..

[20] Elzwawy A., Piskin H., Akdogan N., Volmer M., Reiss G., Marnitz L., Moskaltsova A., Gurel O., Schmalhorst J.-M. (2021). Current trends in planar Hall effect sensors: Evolution, optimization, and applications. J. Phys. D Appl. Phys..

[21] Ward L., Agrawal A., Choudhary A., Wolverton C. (2016). A general-purpose machine learning framework for predicting properties of inorganic materials. npj Comput. Mater..

[22] Oezer B., Piskin H., Akdogan N. (2019). Shapeable Planar Hall Sensor With a Stable Sensitivity Under Concave and Convex Bending. IEEE Sens. J..

[23] Firkowska I., Giannona S., Rojas-Chapana J.A., Luecke K., Brüstle O., Giersig M. (2008). Biocompatible nanomaterials and nanodevices promising for biomedical applications. Nanomaterials for Application in Medicine and Biology.

[24] Capar O., Yildirim M., Cinar H., Oksuzoglu R.M. (2009). Influence of deposition technique on growth and resistivity of Ta/NiFe nano films. Acta Crystallogr. A.

[25] Kaya A. (2019). Magnetoresistance Measurement of Thin Film with Hall Effect Technique. Master’s Thesis.

[26] Smith A., Bahl C.R., Bjørk R., Engelbrecht K., Nielsen K.K., Pryds N. (2012). Materials challenges for high performance magnetocaloric refrigeration devices. Adv. Energy Mater..

[27] Horton M.K., Montoya J.H., Liu M., Persson K.A. (2019). High-throughput prediction of the ground-state collinear magnetic order of inorganic materials using density functional theory. npj Comput. Mater..

[28] Zheng C., Zhu K., Freitas S.C., Davies J.E., Eames P., Freitas P., Kazakova O., Kim C.G., Leung C.W., Liou S.H. (2019). Magnetoresistive sensor development roadmap (non-recording applications). IEEE Trans. Magn..

[29] Piskin H., Akdogan N. (2019). Interface-induced enhancement of sensitivity in NiFe/Pt/IrMn-based planar Hall sensors with nanoTesla resolution. Sens. Actuators A Phys..

[30] Talantsev A., Elzwawy A., Kim C.G. (2018). Effect of NiFeCr seed and capping layers on exchange bias and planar Hall voltage response of NiFe/Au/IrMn trilayer structures. J. Appl. Phys..

[31] Henriksen A.D., Rizzi G., Hansen M.F. (2016). Planar Hall effect bridge sensors with NiFe/Cu/IrMn stack optimized for self-field magnetic bead detection. J. Appl. Phys..

[32] Rhone T.D., Chen W., Desai S., Torrisi S.B., Larson D.T., Yacoby A., Kaxiras E. (2020). Data-driven studies of magnetic two-dimensional materials. Sci. Rep..

[33] Hung T.Q., Oh S., Kumar A.S., Jeong J.R., Kim D.Y., Kim C.G. (2009). Optimization of the multilayer structures for a high field-sensitivity biochip sensor based on the planar Hall effect. IEEE Trans. Magn..

[34] Ho T.K. Random decision forests. Proceedings of the 3rd International Conference on Document Analysis and Recognition.

[35] Pedregosa F., Varoquaux G., Gramfort A., Michel V., Thirion B., Grisel O., Blondel M., Prettenhofer P., Weiss R., Dubourg V. (2011). Scikit-learn: Machine learning in Python. J. Mach. Learn. Res..

[36] Jeong W., Kim M., Ha J.H., Zulkifli N.A.B., Hong J.I., Kim C.G., Lee S. (2019). Accurate, hysteresis-free temperature sensor for health monitoring using a magnetic sensor and pristine polymer. RSC Adv..

[37] Maity T., Trodahl H.J., Granville S., Vézian S., Natali F., Ruck B.J. (2020). Magnetoresistance of epitaxial GdN films. J. Appl. Phys..

[38] Raquet B., Viret M., Sondergard E., Cespedes O., Mamy R. (2002). Electron-magnon scattering and magnetic resistivity in 3d ferromagnets. Phys. Rev. B.

[39] Chakraborty S., Tomsett R., Raghavendra R., Harborne D., Alzantot M., Cerutti F., Srivastava M., Preece A., Julier S., Rao R.M. (2017). Interpretability of deep learning models: A survey of results. Proceedings of the 2017 IEEE SmartWorld, Ubiquitous Intelligence & Computing, Advanced & Trusted Computed, Scalable Computing & Communications, Cloud & Big Data Computing, Internet of People and Smart City Innovation (SmartWorld/SCALCOM/UIC/ATC/CBDCom/IOP/SCI).

[40] Chen C.-W. (2013). Magnetism and Metallurgy of Soft Magnetic Materials.

[41] Gilmer J., Schoenholz S.S., Riley P.F., Vinyals O., Dahl G.E. Neural message passing for quantum chemistry. Proceedings of the 34th International Conference on Machine Learning.

[42] Curtarolo S., Setyawan W., Hart G.L., Jahnatek M., Chepulskii R.V., Taylor R.H., Wang S., Xue J., Yang K., Levy O. (2012). Aflow: An automatic framework for high-throughput materials discovery. Comput. Mater. Sci..

[43] Ortiz C., Eriksson O., Klintenberg M. (2009). Data mining and accelerated electronic structure theory as a tool in the search for new functional materials. Comput. Mater. Sci..

[44] Krzyk S., Schmidsfeld A., Klaeui M., Ruediger U. (2010). Magnetotransport effects of ultrathin Ni80Fe20 films probed in situ. New J. Phys..

[45] Pavarini E. (2021). Solving the strong-correlation problem in materials. La Rivista del Nuovo Cimento.

[46] Roy A., Sampathkumar P., Kumar P.S.A. (2020). Development of a very high sensitivity magnetic field sensor based on planar Hall effect. Measurement.

[47] Dzhun I.O., Gerasimenko A.V., Ezhov A.A., Bezzubov S.I., Rodionova V.V., Gritsenko C.A., Chechenin N.G. (2023). Temperature dependence of magnetization dynamics in Co/IrMn and Co/FeMn exchange biased structures. Magnetochemistry.

[48] Kobs A., Oepen H.P. (2016). Disentangling interface and bulk contributions to the anisotropic magnetoresistance in Pt/Co/Pt sandwiches. Phys. Rev. B.

